# Exploring tailor-made Brønsted acid sites in mesopores of tin oxide catalyst for β-alkoxy alcohol and amino alcohol syntheses

**DOI:** 10.1038/s41598-021-95089-1

**Published:** 2021-08-03

**Authors:** Pandian Manjunathan, Varsha Prasanna, Ganapati V. Shanbhag

**Affiliations:** Materials Science and Catalysis Division, Poornaprajna Institute of Scientific Research (PPISR), Bidalur, Devanahalli, Bengaluru, Karnataka 562164 India

**Keywords:** Catalyst synthesis, Catalytic mechanisms, Heterogeneous catalysis

## Abstract

The generation of Brønsted (Sn–OH) and Lewis (coordinatively unsaturated metal centers) acidic sites on the solid surface is a prime demand for catalytic applications. Mesoporous materials are widely employed as catalysts and supports owing to their different nature of acidic sites. Nevertheless, the procedure adopted to generate acid functionalities in these materials involves tedious steps. Herein, we report the tunable acidic sites containing Brønsted sites with relatively varied acid strength in tin oxide by employing soft template followed by simple thermal treatment at various temperatures. The readily accessible active sites, specifically Brønsted acidic sites distributed throughout the tin oxide framework as well as mesoporosity endow them to perform with exceptionally high efficiency for epoxide ring opening reactions with excellent reusability. These features promoted them to surpass stannosilicate catalysts for the epoxide ring opening reactions with alcohol as a nucleophile and the study was extended to aminolysis of epoxide with the amine. The existence of relatively greater acid strength and numbers in T-SnO_2_-350 catalyst boosts to produce a high amount of desired products over other tin oxide catalysts. The active sites responsible in mesoporous tin oxide for epoxide alcoholysis were studied by poisoning the Brønsted acidic sites in the catalyst using 2,6-lutidine as a probe molecule.

## Introduction

The high surface area materials containing tunable porosity are known to be interesting and very promising in catalysis. They offer better catalytic performance as heterogeneous catalysts than their nonporous counterparts in various organic reactions owing to their properties like high surface area, nanocrystalline nature and pore volume^1,2^. Importantly, employing solid materials as catalysts has attracted significant attention due to their potential in producing a variety of value-added platform chemicals. In addition, mesoporous materials are desired as catalysts due to their structural stability, flexibility in tuning acidic properties namely number, nature (Brønsted or Lewis), and relative strength of acid sites^3,4^. Generally, the acidic sites in these materials are either created by grafting of mercaptosilane on the internal pore surface and followed by oxidation or by a charge imbalance in the framework structure^5–7^. However, the procedure adopted to generate acid functionalities in these materials involves tedious steps.

Metal oxides invariably find applications as catalyst supports owing to their acidic, basic or redox properties. Unlike mixed metal oxides, the individual metal oxide is not well established as catalyst. Traditionally, the metal oxides have been widely employed in adsorption, separation and energy conversion. In general, the surface of metal oxides consists of oxide (O^2−^) ions, coordinatively unsaturated cations (M^n+^) as Lewis acid sites and the cations terminated by –OH groups (M-OH) as Brønsted acid sites that are available unless treated at elevated temperatures^3,8^. These M-OH groups are formed by reducing the coordinatively unsaturated cations through dissociative adsorption of H_2_O molecules^9–12^.

Mesoporous materials are widely employed as catalysts, and the nature of acidic sites have been identified and thoroughly characterized^13,14^. In addition, the metal oxides possessing mesopores (pore size between 2 and 50 nm) can offer better catalysis as they share the diverse properties like porosity and acidity originated from the characteristics of mesoporous materials and metal oxides. The acid sites in these materials are either the Brønsted (hydroxyl groups attached to the metal center) or Lewis (electron-deficient metal centers)^1,15^. The number, nature and relative strength of acidic sites in these materials can be tuned by employing different synthetic strategies including the employ of soft template and calcination temperature.

**Scheme 1:**
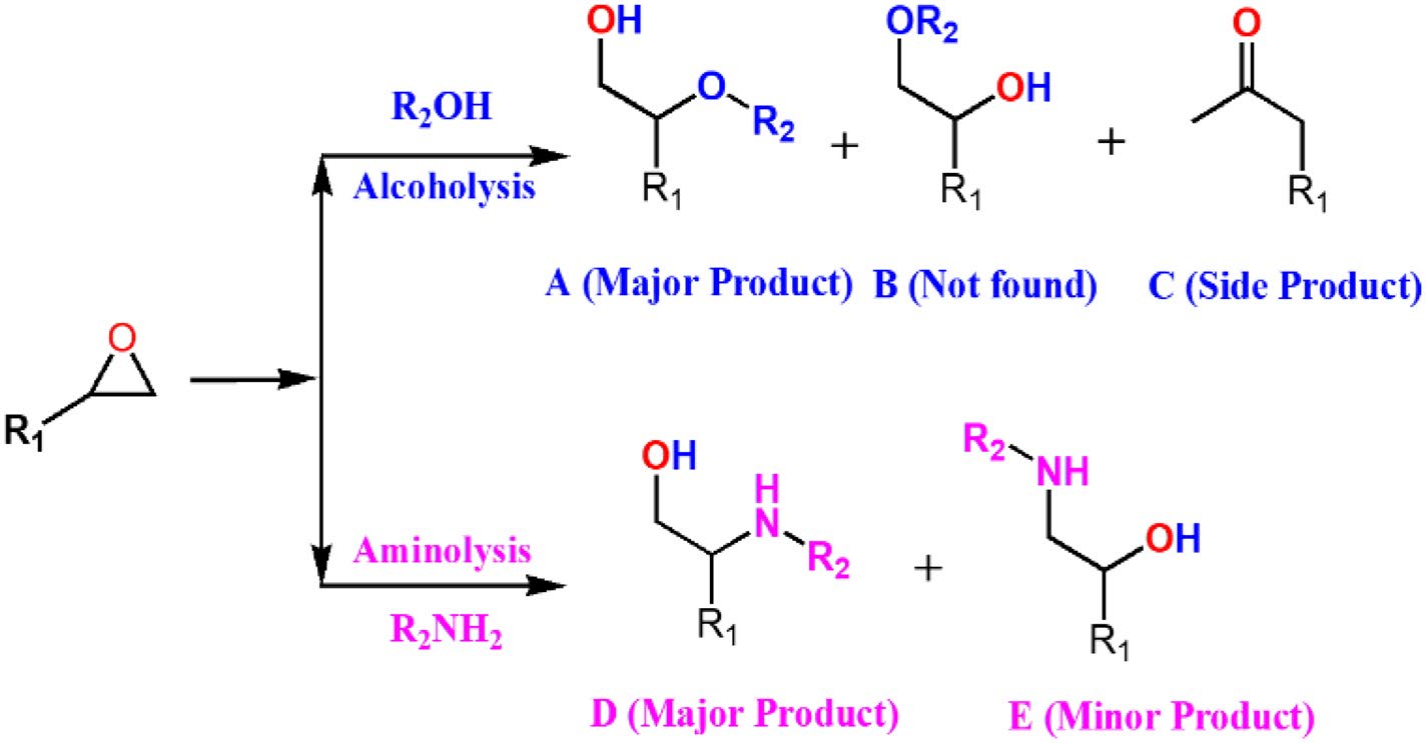
Ring-opening reaction of epoxide with alcohol and amine.

Epoxides constitute important intermediates to produce fine and bulk chemicals^3,16^. The ring-opening reaction of epoxides with nucleophiles like alcohols and amines produce β-alkoxyalcohols and β-amino alcohols respectively (Scheme 1)^17–20^ which have tremendous applications as intermediates in pharmaceuticals. β-Alkoxy alcohols are the versatile intermediates to synthesize many organic compounds in making a wide range of unnatural amino acids, biologically active and synthetic. The epoxide ring opening reaction by alcohols is an important route to produce β-alkoxy alcohols. However, alcohols possess relatively low nucleophilicity, and hence, they require catalyst possessing stronger acidity or basicity to enhance reactivity for alcoholysis of epoxides^21,22^. They are conventionally synthesized by using homogeneous catalyst. However, homogeneous catalysts majorly suffer from poor catalyst recovery, tedious product purification and reactor corrosion which makes the process environmentally unfriendly.

Heterogenous catalysts are more desirable for these reactions as it avoids the intrinsic shortcomings of homogeneous catalytic systems. In recent years, various acidic catalysts have been developed to synthesize β-alkoxyalcohols and β-aminoalcohols. For instance, alcoholysis of epoxides was studied over a series of metal–organic framework catalysts containing different nature of acidic sites containing Brønsted and Lewis^21,22^. The presence of Brønsted acidic sites in MIL-101-Cr-SO_3_H showed a better performance over the pristine MIL-Cr-101 containing Lewis acid sites which suggests the importance of Brønsted acidity^21^. The Fe(BTC)^21^ and Cr-MIL-101 encapsulated Keggin phosphotungstic acid^23^ showed good catalytic activity to produce high yield of β-alkoxyalcohols. However, it utilized a high catalyst amount and very high alcohol concentration which acts as a reactant (nucleophile). Moreover, the catalyst failed to retain its activity and it showed ~ 40% decrease in activity during recycle and which confirms the instability of the catalyst upon reuse^23^. Also, various heterogeneous catalysts namely amberlyst-15, aluminosilicates, metal–organic framework, silica, polymer stabilized metal complexes and more have been reported for epoxide ring opening reactions^19–27^. However, the current process often suffers from prolonged reaction time, drastic reaction conditions, unsatisfactory conversion and poor product regioselectivity^23^.

Our earlier investigation on mesoporous tin oxide as catalyst inspired us to further explore the catalytic applications in other important organic transformations^3^. Herein, we report the catalytic efficiency of mesoporous tin oxide in solvent free ring opening of epoxides with alcohols and amines to synthesize β-alkoxy alcohol and β-amino alcohols. A few conventional heterogeneous catalysts such as stannosilicates and aluminosilicate were also studied under the same reaction conditions to identify the impact of acidic properties. Importantly, tin oxide as catalyst requires no grafting of active centre in the catalyst support and therefore considered to overcome the tedious steps involved in introducing acidic group functionalities without the risk of pore blockage or leaching of grafted component. Also, the tin oxide is not exploited to a greater extent as a catalyst in heterogeneous catalysis research.

## Experimental

### Chemicals and materials

Cetyltrimethylammonium bromide (CTAB), tin chloride pentahydrate, 1-propyl amine, 1-butyl amine, propylene oxide and epichlorohydrin were procured from Loba Chemie Pvt. Ltd, India. Ammonium hydroxide (25 wt%), aniline, cyclohexanol and 1-butanol were obtained from Merck Pvt. Ltd, India. Styrene oxide, methanol, 2-methoxy-2-phenylethanol, ethanol, 1-propanol, propylene oxide, cyclohexene oxide, cyclooctane oxide, epichlorohydrin, phenyl acetaldehyde, tetraethylorthosilicate and pluronic P123 were procured from Sigma-aldrich. NH_4_-beta (SAR, SiO_2_/Al_2_O_3_ = 25) was obtained from Nankai University Catalyst Co., [H^+^ form of beta (H-beta) was obtained by calcining at 540 °C for 4 h.

### Synthesis of mesoporous tin oxide

The mesoporous tin oxide catalysts were synthesized by following our previous reported procedure^3^. In a typical synthesis, 18 g of cetyltrimethylammonium bromide (as soft template) was added into 150 ml distilled water and was constantly stirred at 30 °C to attain a homogeneous solution. To the above solution, 12 ml of NH_4_OH diluted with 48 ml of distilled H_2_O was added with constant stirring. Later, the aq. SnCl_4_ solution containing 15 g of SnCl_4_·5H_2_O in 150 ml of H_2_O was added slowly with constant stirring to get white slurry and stirred for 3 h and further aged for 48 h at room temperature. Later, it was filtered, washed and dried at 100 °C. Then the obtained solid material was crushed into powder and calcined at 300, 350, 400 and 500 °C under flowing air with the heating rate of 2 °C min^−1^ for 2 h, and samples were labelled as T-SnO_2_-x (where x = calcination temperature and T = template assisted synthesis). In addition, the tin oxide was synthesized in the absence of cetyltrimethylammonium bromide by employing the above-mentioned procedure. The obtained material was calcined at 350 °C for 2 h and labelled as TF-SnO_2_-x (where TF = template free synthesis).

Sn-SBA-15 was prepared according to the literature^7^. In a typical synthesis, 2 g of Pluronic P123 was dissolved in 60 ml of 2 M HCl and 15 ml of distilled H_2_O. To the above solution, a calculated amount of SnCl_2_·2H_2_O (Si/Sn = 20) was added and the mixture was stirred at 40 °C for 3 h. Then, 4.25 g of tetraethylorthosilicate was added dropwise to the above mixture and continued stirring for 24 h. Later, the resulting mixture was hydrothermally treated at 100 °C for 24 h. Then, the mixture was filtered, washed and dried at 100 °C for 12 h, and calcined in air at 550 °C for 6 h.

The detailed experimental procedures for all the characterization techniques used in this study are provided in the Supporting information.

### Catalyst evaluation

The catalyst evaluation for epoxide ring opening reactions was performed in a round-bottom glass flask fitted with a reflux condenser. The glass reactor was charged with the reactants and catalyst, and stirred at a desired temperature using magnetic stirrer with hot plate. The progress of the reaction and the product analysis was examined by using Agilent 7890B gas chromatography equipped with a DB-WAX capillary column (0.25 mm I.D and 30 m length) and flame ionization detector.

### Procedure for the chemisorption of 2,6-lutidine in T-SnO_2_-350 catalyst

Prior to the chemisorption, the T-SnO_2_-350 catalyst was pretreated at 350 °C for 1 h, then cooled to 250 °C and later placed in a desiccator. Then, the catalyst was saturated with 2,6-lutidine and heated for 1 h at 150 °C to remove physisorbed molecule. Later, it was employed in the reaction medium as 2,6-lutidine-treated-T-SnO_2_-350 catalyst.

## Results and discussion

### Catalyst characterization

Low angle XRD pattern of T-SnO_2_-300 (calcined at 300 °C) showed a diffraction peak at low angle ascribed to the 2-dimensional hexagonal structure (shown in ESI Fig. S1). Nonetheless, the long-range order in mesopores are not retained for the T-SnO_2_-x calcined at higher calcination temperatures ≥ 350 °C indicated by the absence of diffraction peaks at low angle. The wide angle XRD patterns of tin oxide calcined at different temperatures showed diffraction peaks that are assigned to P42/mnm space group of tetragonal rutile crystal structure (ESI Fig. S1). Compared to T-SnO_2_-300 (tin oxide calcined at 300), a narrowing of diffraction peaks was observed with increasing of calcination temperature ≥ 350 °C. The peak narrowing suggests the gradual increase in crystallinity attributed to the agglomeration of crystallites at higher temperatures. Meanwhile, the crystallite size in tin oxides was calculated from Scherrer equation that showed a gradual increase in crystallite size to 13.8 nm from 4.4 nm with increasing of calcination temperatures ranging from 300 to 500 °C (Table S1). Notably, the TEM images (ESI Fig. S2) of T-SnO_2_-350 shows a wormhole like topology with the mesostructure is constructed by nanocrystalline domains and which fairly agrees with the Scherrer calculation. HR-TEM image of T-SnO_2_-350 confirms a well-defined lattice fringes showing a typical characteristic of SnO_2_ in tetragonal rutile phase as shown in ESI Fig. S2d.

**Figure 1:**
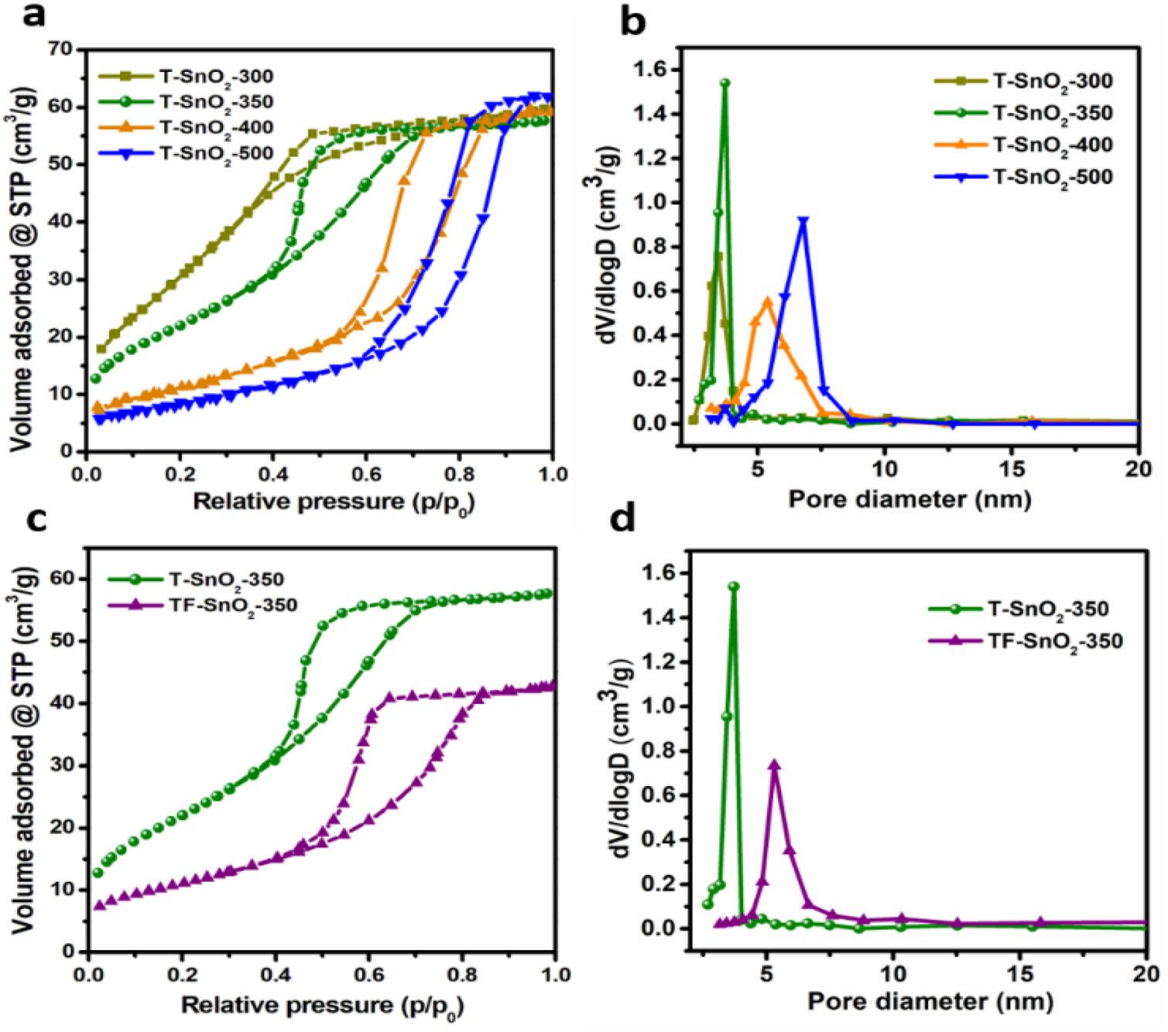
(**a**,**c**) Nitrogen sorption isotherm and (**b**,**d**) pore size distribution of SnO_2_ catalysts. (Reproduced from reference^3^).

Figure 1 shows the corresponding N_2_ isotherms and BJH pore size distributions, which reveals all the tin oxide catalysts exhibit Type IV isotherm, confirms the characteristic of mesoporous materials. Notably, the hysteresis loop for tin oxide changes from H2 to H1 type as the calcination temperature increases ≥ 400 °C. The T-SnO_2_-x (template assisted) calcined at 300 and 350 °C gave H2 kind of hysteresis which corresponds to ink bottle like pores. It is designated to the spherical type pores in addition to cylindrical pores in the catalyst^28^. However, T-SnO_2_-400 and 500 exhibited H1 like hysteresis corresponding to cylindrical-type pore channels in porous materials. The BJH pore size distribution confirms the presence of mesoporosity in tin oxides and reveals the systematic increase in pore size diameter from 3.4 to 6.7 nm by enhancing calcination temperature by 50 °C starting from 300 to finally reach 500 °C. Among the mesoporous tin oxide catalysts, the T-SnO_2_-300 showed a greater surface area of 160 m^2^ g^−1^. However, further increasing of calcination showed a significant decline in surface area to the lowest of 51 m^2^ g^−1^ for 500 °C. It could be attributed to the pore damage at higher calcination temperatures^3,29,30^. Interestingly, the TF-SnO_2_-350 (template-free) also contained mesoporosity with the average pore size of 5.3 nm indicated by type IV isotherm and H2-type hysteresis. Nevertheless, the surface area and pore volume of TF-SnO_2_-350 was lower than that of template assisted T-SnO_2_-350 suggesting the benefit of employing CTAB as soft template during the synthesis. The results obtained from CHNS analysis confirm the absence of carbon and nitrogen (below detection limits of 0.01%) in the calcined samples which indicates the complete removal of soft template during calcination step.

The nature, number and strength of acidic sites in tin oxide catalysts were examined by Py-FTIR, NH_3_-TPD and ^1^H-MAS NMR techniques, respectively. Pyridine FTIR study (shown in ESI Fig. S3) reveals the presence of different nature of acidic sites namely Brønsted (1540 cm^−1^) and Lewis (1450 cm^−1^)^31^ with variable concentrations (ratio). Importantly, the SnO_2_ catalysts showed greater number of Brønsted acid sites compared to Lewis. The existence of Brønsted sites is due to the surface ‒OH or H‒bonded Sn–OH sites, whereas Sn^4+^ sites in the framework contribute to Lewis acidity. Interestingly, increasing of calcination temperature in SnO_2_ results in systematic decline of B/L ratio due to loss of ‒OH groups by dehydroxylation. Also, the pyridine-FTIR of stannosilicate and aluminosilicate (H-Beta) were investigated, and it showed (ESI Fig. S3) greater amount of Lewis acid character compared to SnO_2_ catalysts. The FTIR of SnO_2_ catalysts exhibited peaks at ~ 3410 and ~ 1620 cm^−1^ corresponding to the stretching and bending vibrations of –OH (hydroxyl) groups, respectively (ESI Fig. S4). At lower calcination temperature of 300 and 350 °C, the intensity of the peaks were similar, whereas it decreased with increase in calcination temperature > 350 °C. Abundant surface –OH groups with tunable acidic sites and their strength make the SnO_2_ catalyst a better solid acid catalyst for the alcoholysis and aminolysis reactions.

The number of acidic sites in tin oxide was confirmed from NH_3_-TPD (Table 1, ESI Fig. S5) and the measurements were performed till its calcination temperature as catalyst may give peaks unrelated to NH_3_ desorption at higher temperatures causing erroneous result. Among the tin oxide catalysts, T-SnO_2_-350 gave a higher number of acidic sites containing 440 μmol g^−1^, whereas the acidity of tin oxides ranging from 440 to 240 μmol g^−1^ with varied calcination temperatures. Notably, the total number and Brønsted acid sites in T-SnO_2_-x decreases with increase in the calcination temperatures >350 °C attributed to the dehydroxylation of surface Sn–OH moieties which results in –Sn–O–Sn– yielding SnO_2_ containing lower –OH moiety which acts as Brønsted acidic sites. Notably, the template free TF-SnO_2_-350 contains acidity of 300 μmol g^−1^, a lower number of acidic sites than the soft template assisted T-SnO_2_-350. The lower acidity in TF-SnO_2_-350 could be due to its lower surface area. Although the number of acidic sites in SnO_2_ catalysts were confirmed from NH_3_-TPD, the acid strength in these catalysts were not clearly understood due to the decomposition of –OH moieties beyond its calcination temperature resulting in the formation of H_2_O molecules which merges with signal obtained for desorbed NH_3_ during analysis.

**Table 1:** Textural and chemical properties of tin oxide and other solid acid catalysts.

Catalyst	T-SnO_2_-300^a^	T-SnO_2_-350^a^	T-SnO_2_-400^a^	T-SnO_2_-500^a^	TF-SnO_2_-350^a^	H-Beta^a^	Sn-SBA-15
S_BET_ (m^2^ g^−1^)	160	105	55	51	50	485	820
Pore volume (cm^3^ g^−1^)	0.12	0.11	0.10	0.10	0.06	0.44	1.22
Pore size (nm)	3.4	3.8	5.4	6.8	5.3	^b^	4.1
B/L ratio	4.4	4.0	2.9	1.8	3.7	3.8	0.4
Acidity (μmol NH_3__des_ g^−1^)	410	440	290	240	300	1500	380
Brønsted acidity (μmol NH_3__des_ g^−1^)	334	**352**	216	154	236	1188	109
Lewis acidity (μmol NH_3__des_ g^−1^)	76	88	74	86	64	313	271

^a^Reference^3^, ^b^0.66 × 0.67 nm and 0.56 × 0.56 nm.

**Figure 2:**
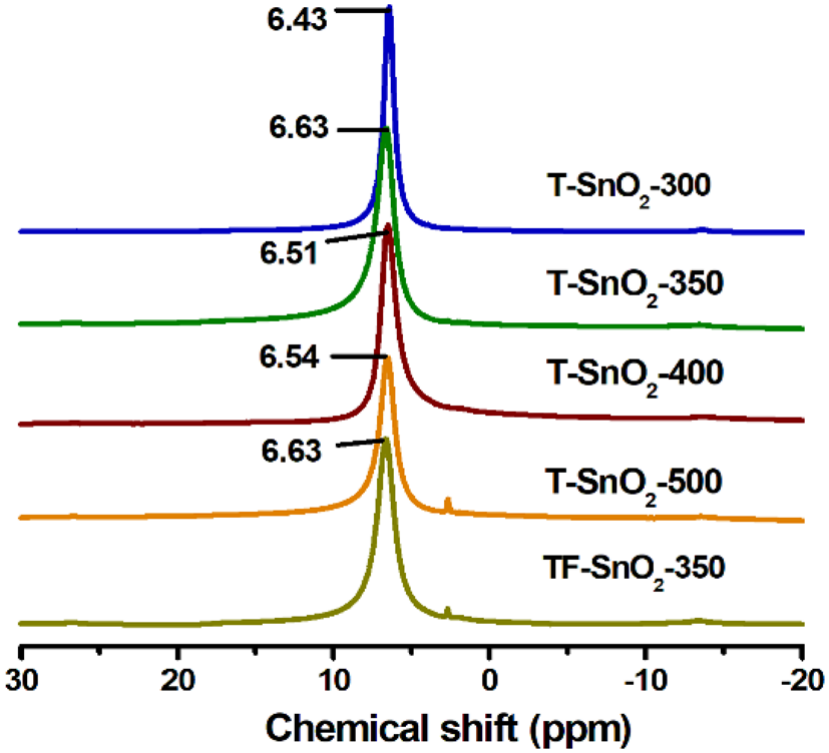
^1^H MAS NMR of SnO_2_ catalysts. (Reproduced from reference^3^).

In order to further investigate, ^1^H MAS NMR of tin oxides was studied to determine the relative acidic strength from the deviation in chemical shift which occurs because of bond polarization^32,33^. An increment in chemical shift from 6.43 to 6.63 ppm was observed due to increase in calcination from 300 to 350 °C (Fig. 2), where the low-field shift indicates an increase of strength of acidity in the catalyst calcined at 350 °C. In ^1^H MAS NMR, the change in chemical shift may be due to weak H-bonding interaction in Sn‒O‒H moieties. Higher the low-field movement of chemical shift, greater is the strength of acidic sites, caused by bond polarization in Sn‒O‒H groups. Importantly, the acidic strength in tin oxide catalyst decreases by increasing the calcination temperature > 350 °C as confirmed by the chemical shift towards high-field. The chemical shift of proton was identical for both template-free and templated tin oxide catalysts (Fig. 2) which indicated the similarity in acidic strengths in both the catalysts.

## Catalytic activity studies

### Alcoholysis of epoxy styrene with methanol

**Scheme 2:**
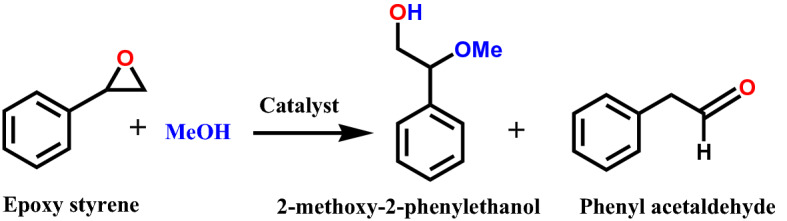
Reaction scheme for alcoholysis of epoxy styrene with methanol.

To begin with, epoxy styrene was selected for ring opening reaction with methanol as a nucleophile to achieve greater yield of the desired product (Scheme 2). The ring opening reaction of epoxy styrene with methanol yielding 2-methoxy-2-phenylethanol with different catalysts is shown in Table 2. Only a trace conversion was observed in absence of catalyst even reaction after 1 h. On the other hand, catalysts with different properties were tested under identical number of acidic sites to identify the most active one for the reaction. Among them, T-SnO_2_-350 gave the best catalytic performance under similar conditions with a greater yield to 2-methoxy-2-phenylethanol. The T-SnO_2_-350 containing high amount of Brønsted acidic sites was found be to very active and provided a greater catalytic activity for ring opening of epoxy styrene with methanol. Notably, T-SnO_2_-350 gave higher initial rate of formation of 700 mol·(mol_acidity_ _taken_·h)^−1^ for 2-methoxy-2-phenylethanol compared to other catalysts screened in this work. Under identical number of acidic sites, the Sn-SBA-15 containing higher Lewis acid sites showed poor activity toward the reaction, which suggests the importance of Brønsted acidic sites for the reaction. Strikingly, TF-SnO_2_-350 containing similar acidic strength (chemical shift = 6.63 ppm) under identical amount of acidic sites showed lower activity with epoxy styrene conversion of 45.8% compared to T-SnO_2_-350 due to the presence of lower surface area. Notably, H-beta zeolite, a large pore microporous aluminosilicate converted only 27.2% of epoxy styrene but the activity was greater than that of Sn-SBA-15. This could be attributed to the existence of greater Brønsted acid sites in H-beta containing B/L ratio of 3.8 compared to Sn-SBA-15. A combined investigation employing different variety of catalysts bearing varied acidic natures clearly shows that the Lewis acidity contributed relatively much lower compared to Brønsted acidity in T-SnO_2_-350. A few well-known conventional base catalysts namely MgO and CaO were investigated to further understand the role of acidity in this reaction. It showed only 0.1% conversion which is equivalent to blank run and further confirms that this reaction requires a catalyst possessing acidic characters. In particular, among the catalysts, T-SnO_2_-350 exhibited higher yield for 2-methoxy-2-phenylethanol which can be ascribed to the combination of structural and chemical properties of the catalyst.

**Table 2:** Catalytic activity of different catalysts for alcoholysis of epoxy styrene with methanol.

Catalysts	Acidity (μmol NH_3__des_ g^−1^)^a^	B/L ratio^b^	Time (min)	Epoxy styrene conv. (wt%)	Product Selectivity (wt%)		A yield (wt%)	Rate^e^ [mol·(mol _acidity_ _taken_·h)^−1^]
					A	B		
Blank	–	–	10	< 0.1	100	–	< 0.1	–
Blank	–	–	60	0.1	100	–	0.1	–
TF-SnO_2_-350	300	3.7	10	25.2	91.4	8.6	23.0	523
TF-SnO_2_-350	300	3.7	30	45.8	90.9	9.1	41.6	137
T-SnO_2_350	440	4.0	10	33.1	93.2	6.8	30.8	700
T-SnO_2_350	440	4.0	30	56.0	92.6	7.4	51.9	173
H-beta	1500	3.8	10	17.3	92.8	7.2	16.1	364
H-beta	1500	3.8	30	27.2	93.1	6.9	25.3	84
Sn-SBA-15	380	0.4	10	14.1	95.6	4.4	13.5	306
Sn-SBA-15	380	0.4	30	23.6	95.2	4.8	22.5	75
SBA-15	nd	nd	10	1.3	94.2	5.8	1.2	–
SBA-15	nd	nd	30	2.2	93.4	6.6	2.1	–
CaO^d^	18^c^	nd	10	< 0.1	100	–	< 0.1	55
CaO^d^	18^c^	nd	30	0.1	100	–	0.1	0.3
MgO^d^	13^c^	nd	10	< 0.1	100	–	< 0.1	77
MgO^d^	13^c^	nd	30	0.1	100	–	0.1	0.3

Reaction conditions: epoxy styrene = 2 g, methanol = 5.3 g (mole ratio = 1:10), catalyst = 44 μmoles of acidic sites, reaction temp = 65 °C. ^a^NH_3_-TPD, ^b^Py-FTIR, ^c^basicity (μmol g^−1^)^34^, ^d^catalyst = 0.1 g, nd = not detectable. A = 2-methoxy-2-phenylethanol, B = phenyl acetaldehyde, ^e^Initial rate of product formation (2-methoxy-2-phenylethanol) = mol·(mol_acidity_ _taken_·h)^−1^.

**Figure 3:**
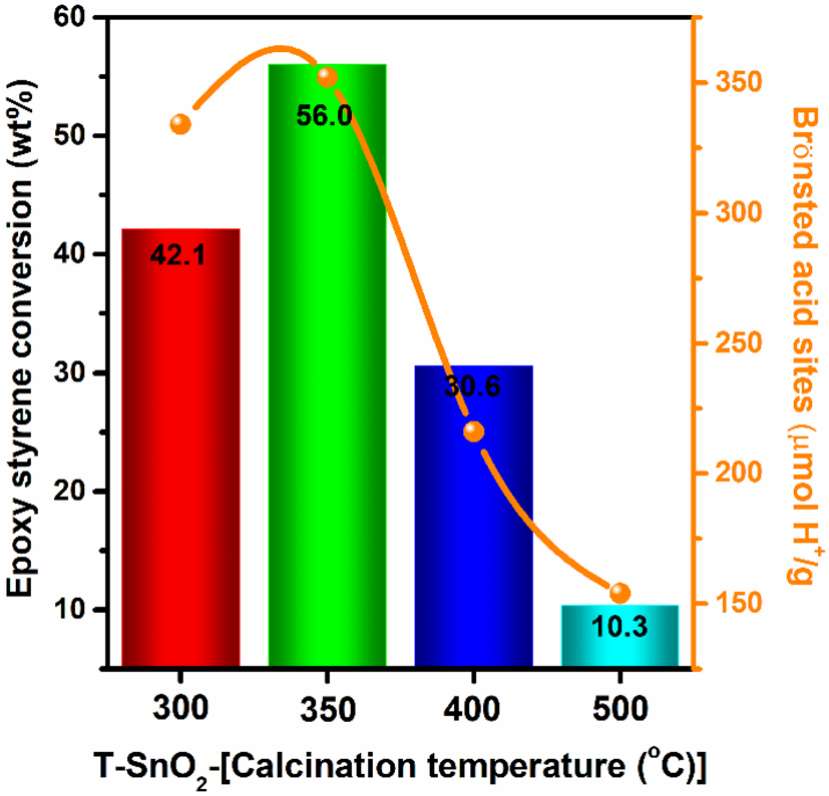
Correlation plot for epoxy styrene conversion v/s SnO_2_ calcination temperature and Amount of Brønsted acidity (μmol H^+^ g^−1^) on alcoholysis of epoxy styrene with methanol. Reaction conditions: epoxy styrene = 2 g, methanol = 5.3 g (mole ratio = 1:10), catalyst = 0.1 g, reaction temp = 65 °C, time = 30 min.

Alcoholysis of epoxy styrene with methanol using T-SnO_2_-x catalyst calcined at various temperatures (300‒500 °C) was investigated and presented in Fig. 3 and ESI Table S2. Although, the T-SnO_2_-300 contains higher B/L ratio of 4.4, it showed only 42% epoxy styrene conversion which is lower compared to T-SnO_2_-350. Among the several catalyst parameters such as surface area, pore size, B/L ratio, amount and nature of acidity, the best correlation was obtained with the amount of Brønsted acidic sites and their strength. The increasing of calcination temperature in T-SnO_2_-x > 350 °C resulted in a systematic decline in activity from 56.0 (T-SnO_2_-350) to 10.3% (T-SnO_2_-500) due to the decrease in number of Brønsted acid sites and their strength (obtained from chemical shift of ^1^H MAS NMR) in the catalyst. The T-SnO_2_-350 was better in terms of required acidity and therefore showed highest catalytic activity among the SnO_2_ catalysts. To further understand the catalytic behavior of T-SnO_2_-350, a reaction under identical number of Brønsted acidic sites (as in T-SnO_2_-300) was performed (shown in Table 3) by varying the amount of the catalyst. The T-SnO_2_-350 catalyst showed a greater activity which could be attributed to the presence of greater acidic strength (obtained from ^1^H MAS NMR) in the catalyst. Hence, this clearly suggests that this reaction needs a greater number of Brønsted acidic sites along with acidic strength in the catalyst. It is well known that SBA-15 containing only ‒OH moieties which is extensively used to graft the functional groups like –SO_3_H and more via condensation process. The SBA-15 catalyst calcined at 500 °C containing large number of ‒OH groups showed only 2.2% of epoxy styrene conversion (Table 2). Interestingly, the activity is lower compared to SnO_2_ (with the same calcination temperature of 500 °C) which showed almost fivefold more activity.

**Table 3:** Comparison of catalytic activity of T-SnO_2_-300 and T-SnO_2_-350 catalysts for alcoholysis of epoxy styrene with methanol under identical amount of Brønsted acidic sites.

Catalysts	Brønsted Acidity (μmol H^+^ g^−1^)	B/L ratio^a^	^1^H (ppm)^b^	Epoxy styrene conversion (wt%)	Product Selectivity (wt%)		A (wt%)
					A	B	
T-SnO_2_-300^c^	334	4.4	6.43	42.1	93.2	6.8	39.2
T-SnO_2_-350^c^	352	4.0	6.63	56.0	92.6	7.4	51.9
T-SnO_2_-350^d^	352	4.0	6.63	50.4	93.0	7.0	46.9

^a^Reaction conditions: epoxy styrene = 2 g, methanol = 5.3 g (mole ratio = 1:10), reaction temp = 65 °C, time = 30 min. ^a^Py-FTIR, ^b1^H MAS NMR, ^c^catalyst = 0.1 g, ^d^identical number of Brønsted acidic sites (33 μmol H^+^ g^−1^) as in T-SnO_2_-300, A = 2-methoxy-2-phenylethanol, B = phenyl acetaldehyde.

**Figure 4:**
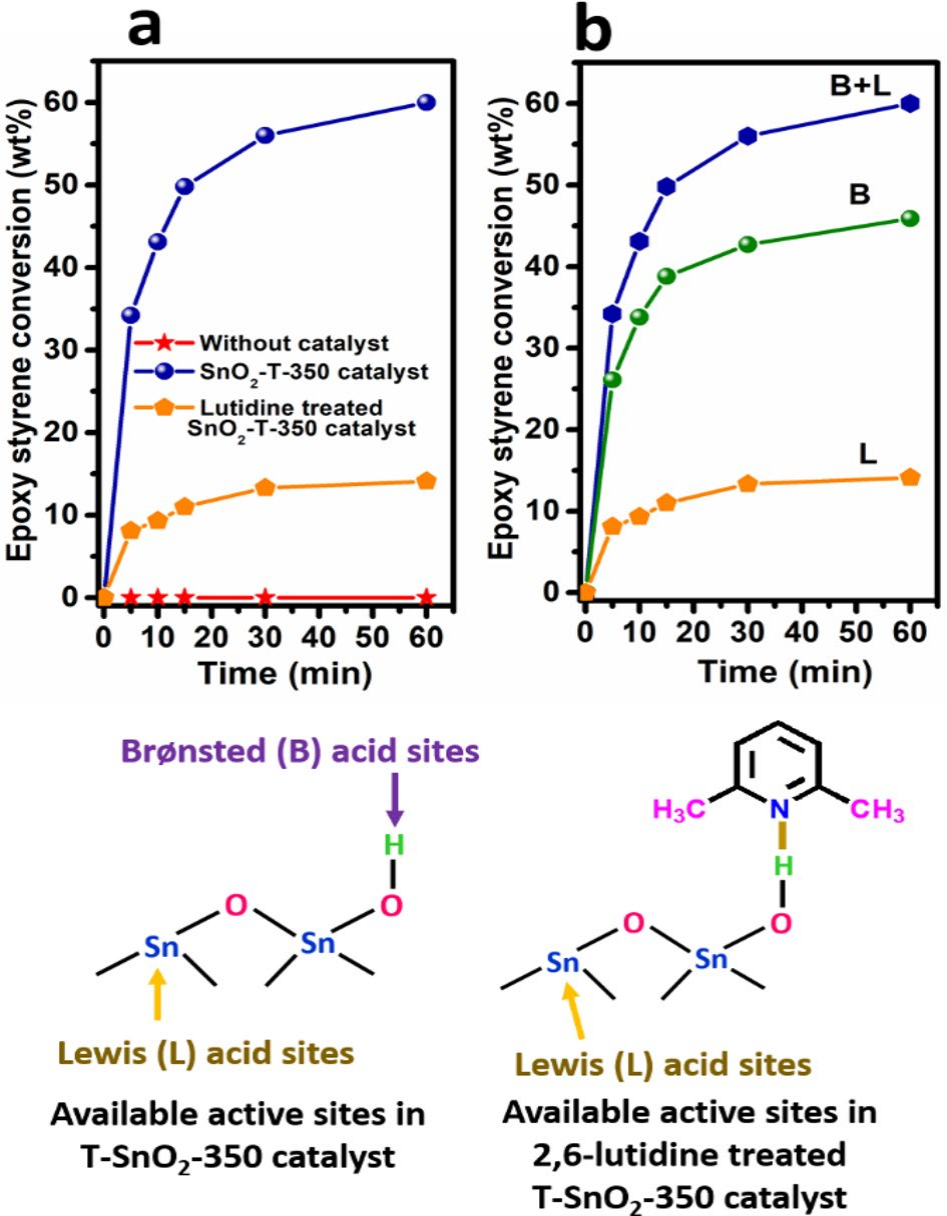
(**a**) Catalytic performance of 2,6-lutidine treated and untreated T-SnO_2_-350 catalysts for alcoholysis of epoxy styrene with methanol. (**b**) Contribution of nature of corresponding active sites responsible for alcoholysis reaction. Reaction conditions: epoxy styrene = 2 g, methanol = 5.3 g (mole ratio = 1:10), catalyst = 0.1 g, reaction temp = 65 °C.

The nature of active sites responsible in T-SnO_2_-350 catalyst for alcoholysis of epoxy styrene was determined by performing a reaction using the catalyst treated with a basic 2,6-lutidine probe molecule. It is known that 2,6-lutidine is a basic molecule which selectively chemisorbs with Brønsted acidic sites but not with Lewis acidic sites due to steric hindrance caused by its methyl groups^35–37^. Therefore, it results in the exposure and participation of only Lewis acidic Sn^4+^ sites during the reaction. Thus, for a 2,6-lutidine-treated-T-SnO_2_-350 catalyst, only 14% epoxy styrene conversion at 60 min was obtained which indicates that the activity was suppressed by blocking the Brønsted acidic sites (Fig. 4a). The untreated active T-SnO_2_-350 catalyst gave 60% epoxy styrene conversion which is significantly higher compared to that of 2,6-lutidine treated-T-SnO_2_-350 catalyst. Figure 4b shows that the contribution of Lewis acidic Sn sites towards the epoxy styrene conversion is negligible (14.1%) compared with that of Brønsted acid sites (45.9%). Therefore, it confirms that both the Lewis (Sn^4+^) and Brønsted acid sites (Sn‒O‒H) in T-SnO_2_-350 catalyst are responsible for this reaction with the major contributions from Brønsted acid sites. Based on these observations, a plausible reaction mechanism is proposed and given in Scheme S1.

The active T-SnO_2_-350 catalyst was further investigated to synthesize a wide range of β-alkoxy alcohols from different epoxides and alcohols. A range of β-alkoxy alcohols was formed with high regioselectivity from epoxy styrene and different alcohols such as methanol, ethanol, 1-propanaol, 1-butanol and cyclohexanol as nucleophiles. As shown in Table 4, the reaction with ethanol and other larger alcohols were progressed slower than with methanol. The epoxy styrene conversion for methanol (100%) was substantially different compared with ethanol (16%), 1-propanol (12%), and 1-butanol (10%) and larger alcohols were less reactive than methanol for 30 min reaction. Further, increasing the reaction time resulted in high product yield for all the alcohols. Apparently, the study of other epoxides namely propylene oxide, epichlorohydrin and cyclohexene oxide with methanol yielded respective β-alkoxy alcohols in high yields at longer reaction time compared to epoxy styrene.

**Table 4:** Ring opening reaction of epoxides with various nucleophiles catalyzed by SnO_2_-T-350.

Epoxide	Nucleophile	Temp (°C)	Product	Time (h)	Conversion (wt%)	Selectivity (wt%)
		Reflux		0.5^b^	100.0	96.5
		Reflux^a^		0.5	16.0	98.0
				8	93.4	97.4
		Reflux^a^		0.5	12.0	100
				8	94.4	100
		Reflux^a^		0.5	10.0	100
				8	97.1	100
		Reflux^a^		1	52.6	83.6
				10	100.0	80.0
		Reflux^a^		1	26.2	45.7
				21	98.6	47.8
		Reflux^a^		1	31.0	100
				21	74.2	100
		Reflux^a^		22	27.2	97.5
		Reflux^a^		1^c^	76.2	87.6
				1^d^	98.7	88.6
		90^c^		1	44.7	22.7
				3	100.0	27.0
		90^c^		1	40.6	13.1
				3	100.0	23.3
		90^c^		1	47.0	100.0
				10	85.0	100.0

Reaction conditions: ^a^epoxide (16.6 mmol), alcohols (500 mmol), catalyst (SnO_2_-T-350) (0.40 g), reaction temp = reflux temp; ^b^catalyst (0.15 g); ^c^amine (20 mmol), epoxide (20 mmol), catalyst (T-SnO_2_-350) (0.12 g); ^d^catalyst (T-SnO_2_-350) (0.48 g).

Besides using alcohols as nucleophiles, the reactivity of amines with epoxy styrene was determined to yield β-amino alcohols via aminolysis (Table 4). The aminolysis of epoxy styrene with aniline at equimolar ratio resulted in moderate epoxy styrene conversion of 76% with 87% selectivity to desired product in 1 h time. Also, both conversion and selectivity increased upon increasing of catalyst weight. Increasing of catalyst amount from 5 to 20 wt% showed a significant increase of epoxy styrene conversion from 76.2 to 98.7% due to an increase in the amount of surface acid sites accessible to the reactant molecules. The reaction of epoxy styrene with aliphatic amines namely propyl amine and butyl amine exhibited 100% conversion but the selectivity for the desired product was low (< 25%) in 3 h reaction time.

Among the amine substrates investigated, the aniline was the most active one, achieving 76.2% epoxy styrene conversion with 87.6% β-amino alcohol selectivity. But, in contrast to aniline, an inadequate regioselectivity was observed for aliphatic primary amines, such as n-propylamine and n-butylamine. Considering the pKa of amines as 4.6 (aniline), 10.2 (n-propyl amine) and 10.6 (n-butyl amine), it showed a decrease in epoxy styrene conversion (from 76.2 to 40.6%) with increasing pKa of amines (from 4.6 to 10.6). The decrease in epoxy styrene conversion could be attributed to the strong adsorption of substrate on the acid sites when amine possessing higher pKa value was employed, thus reducing their capability to activate epoxide molecules. Importantly, the fused cyclic epoxide, cyclohexene oxide showed 85% of conversion with complete selectivity (100%) to the corresponding β-amino alcohols. These results prove that T-SnO_2_-350 is a versatile catalyst for ring-opening of various epoxides to synthesize β-alkoxy alcohols and β-amino alcohols in high yields.

To verify the heterogenous nature of T-SnO_2_-350 catalyst, a hot filtration test was performed. For leaching study, the reaction was stopped after 5 min, and the catalyst in reaction mixture was removed by centrifugation (shown in ESI Fig. S8a). Later, the filtrate was allowed to undergo reaction without a catalyst which showed no further improvement in epoxy styrene conversion which inferred that the reaction cannot occur without a catalyst and the T-SnO_2_-350 catalyst is truly heterogeneous.

**Figure 5:**
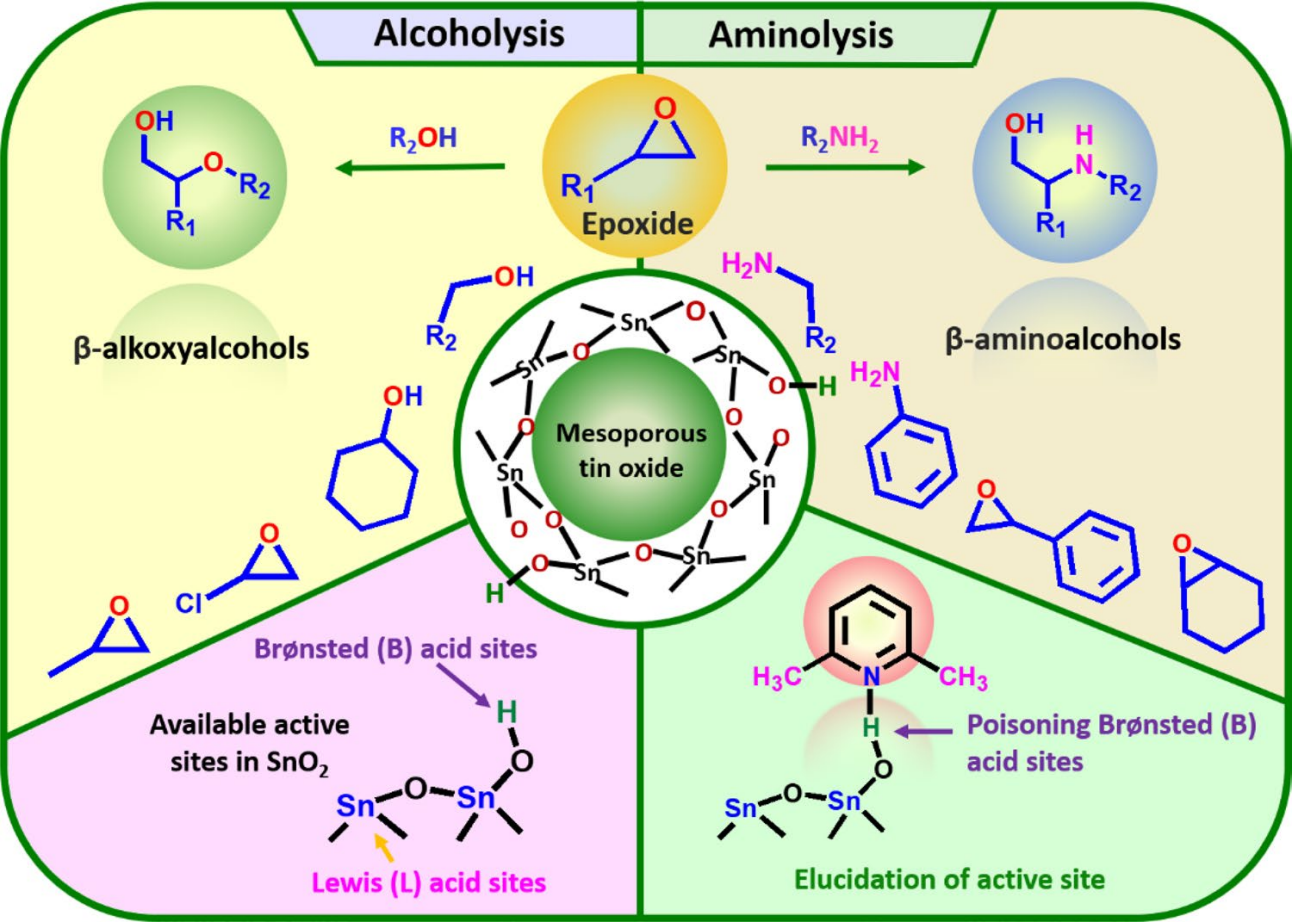
Graphical representation of alcoholysis and aminolysis reactions of epoxides catalyzed by mesoporous tin oxide.

The most active T-SnO_2_-350 catalyst for alcoholysis of epoxy styrene reaction was examined for its reusability with methanol and the results are presented in ESI Fig. S6b. The reaction was studied under optimized conditions for 5 cycles. After each cycle, the catalyst was separated by filtration and washed adequately with methanol to eliminate adsorbed impurities on the catalyst surface. The catalyst was dried at 100 °C for 2 h and later calcined at 350 °C for 2 h under flowing air. The catalyst exhibited a good reusability with a marginal decrease in epoxide conversion (~ 3%) with similar selectivities. Notably, the T-SnO_2_-350 catalyst showed excellent catalytic activity compared to acidic aluminosilicate^21^, MIL-101-Cr-SO_3_H^21^, Fe(BTC)^22^ and Cr-MIL-101 encapsulated Keggin phosphotungstic acid^23^ and few others. The tedious synthesis methodology adopted in these reported catalysts and prolonged reaction time, limits its application in catalysis. In the present work, T-SnO_2_-350 exhibited good stability and reusability without any significant loss in catalytic activity and thus, it provides exceptional properties for potentially wide applications in catalysis (Fig. 5).

## Conclusions

We performed a comprehensive assessment on easier synthesis of porous tin oxide and expandable variants in them which offers tailored properties different from conventional mesoporous catalysts in many characteristics, such as tunable structure (i.e., pore diameter and pore volume) and chemical properties by simple thermal treatment. Employing CTAB as a soft template greatly influenced the structural and chemical properties of SnO_2_. Herein, meso tin oxide, featuring mesoporosity replete with Brønsted acidic –OH sites and Lewis acidic Sn^4+^, has shown exceptionally greater catalytic performance (high activity, excellent selectivity and recyclability) for epoxide ring opening reactions. Moreover, the active site responsible for catalytic activity was identified by 2,6-lutidine studies and it revealed that both the Lewis and Brønsted acid sites are responsible for this reaction with the major contribution from Brønsted sites. Importantly, the soft template assisted T-SnO_2_ catalyst outperformed over other conventional porous aluminosilicates and metallosilicates such as H-Beta and Sn-SBA-15 catalytic materials. The strategy encountered to generate Brønsted acidic sites in SnO_2_ studied in this work is easier and novel compared to functionalization by post-synthesis involving tedious steps. Importantly, soft template assisted SnO_2_ catalyst showed enhanced catalytic performance and proved to be an excellent catalyst for different epoxides with alcohol and amine which opens the ring smoothly and regioselectively to give good yields.

## Supplementary Information

Supplementary Information 1.
