# Supplementary material for: Exploring tailor-made Brønsted acid sites in mesopores of tin oxide catalyst for β-alkoxy alcohol and amino alcohol syntheses
Source: Sci Rep. 2021 Aug 3;11:15718. doi: 10.1038/s41598-021-95089-1 (PMC8333069; doi:10.1038/s41598-021-95089-1)
Supplement: Supplementary file 1 — Supplementary Information 1. [file 41598_2021_95089_MOESM1_ESM.doc]

**Supporting Information**

**Exploring tailor-made Brönsted acid sites in mesopores of tin oxide for β-alkoxy alcohol and amino alcohol syntheses**

Pandian Manjunathan*, Varsha Prasanna and Ganapati V. Shanbhag*

*Materials Science and Catalysis Division, Poornaprajna Institute of Scientific Research (PPISR), Bidalur Post, Devanahalli, Bengaluru-562164, Karnataka, India*

**E-mail addresses: manjunathanp@poornaprajna.org,* [*shanbhag@poornaprajna.org*](mailto:shanbhag@poornaprajna.org)

1. Experimental section & characterization techniques….......................…….S2

2. Fig. S1 .………………………………………............................………….S3

3. Fig. S2…………………………………...................................................…S4

4. Fig. S3..…………………………....................................................……… S4

5. Fig. S4..…………………………....................................................……… S5

6. Fig. S5..…………………………....................................................……… S5

7. Fig. S6..…………………………....................................................……… S7

8. Fig. S7..…………………………....................................................……… S8

9. Fig. S8..…………………………....................................................……… S9

10. Table S1…………………........................................................……………S9

11. Table S2…………………........................................................……………S10

12. Scheme S1…………………........................................................…………S10

13. References………………….............................................................………S11

**S1. General information**

**Characterization techniques**

The phase purity of catalysts was examined by X-ray diffractometer (Bruker D2 phaser) using CuKα radiation with high resolution Lynxeye detector. Mean crystallite size of tin oxide catalysts was calculated by Scherrer equation, D = kλ/βcosθ; D is the mean crystallite size, λ= X-ray wavelength (1.5418 Å); k = 0.94, θ is the Bragg angle (in degrees) and β is the full width at half maximum intensity of the (110) plane of tin oxide. The surface area, pore volume and pore-size distribution of catalysts were determined by nitrogen sorption measurements at 77 K using a Belsorp mini II instrument. The nature of acid sites in catalysts viz, Brønsted and Lewis acidic sites were investigated by pyridine chemisorption study using FT-IR [[^1^](#_ENREF_1)]. The self-supported wafer of catalyst was thermally treated at respective calcination temperature of catalyst for 1 h, then cooled to 250 °C and later placed in a desiccator. Then the catalysts were saturated with pyridine and heated for 1 h at 150 °C to remove the physisorbed pyridine. FTIR spectra of catalysts were recorded in absorbance mode with the wavenumber ranging from 1400 to 1600 cm^–1^. The resultant peaks only due to pyridine–acid interaction in catalysts were obtained by subtracting the spectrum of pyridine untreated catalysts from that of pyridine treated. In the difference spectrum, the peak corresponding to Brønsted acid sites (~1540 cm^-1^) and Lewis acid sites (~1450 cm^-1^) were integrated to obtain the integrated absorbance of respective acid sites. Then, the B/L ratio was determined according to the equations reported by C. A. Emeis [[^2^](#_ENREF_2)].

The Brønsted and Lewis acid sites present in catalysts were quantified by combining the calculated B/L ratio (obtained from Pyridine FTIR) and total acidity (obtained from NH_3_-TPD) [[^3^](#_ENREF_3), [^4^](#_ENREF_4)].

The total amount of available acidic sites in the catalysts were determined by NH_3_-TPD (ammonia-temperature programmed desorption) technique using Belcat II instrument equipped with thermal conductivity detector. In all the experiments, 0.2 g of catalyst was pretreated at its respective calcination temperature for 1 h in a flow of 25 mL/min helium gas at and then cooled to 50 °C. Then, the pretreated catalyst was saturated with 10% NH_3_/He stream (ammonia balanced with helium) at 50 °C for 1 h. The catalyst was flushed by a flow of helium gas for 1 h at 50 °C to remove the physisorbed ammonia in catalyst surface, and then ammonia was desorbed at the temperature ranging from 50 °C to its respective calcination temperature with the heating rate of 10 °C/min. [[^5^](#_ENREF_5)]. The desorption higher than the calcination results in the dehydroxylation peaks leading to an error. Thermogravimetric analysis (TGA) was conducted using NETZSCH TG 209 F1 Libra analyzer under flowing nitrogen. The TEM, HR-TEM images and SAED patterns of catalyst were recorded using TEM-JEOL-2010 instrument. ^1^H MAS NMR spectra of tin oxide catalyst were recorded using Bruker instrument at the ^1^H resonance frequency of 399.78 MHz. The chemical shifts for ^1^H were referenced to tetramethylsilane (TMS).

**Catalyst recycle experiment**

After completion of the experiment, the catalyst was filtered, washed with methanol to remove the adsorbed species on the catalyst surface followed by drying at 100 °C and finally calcined at 350 °C for 2 h. The calcined catalyst was further used for the reaction with a fresh reaction mixture.

.


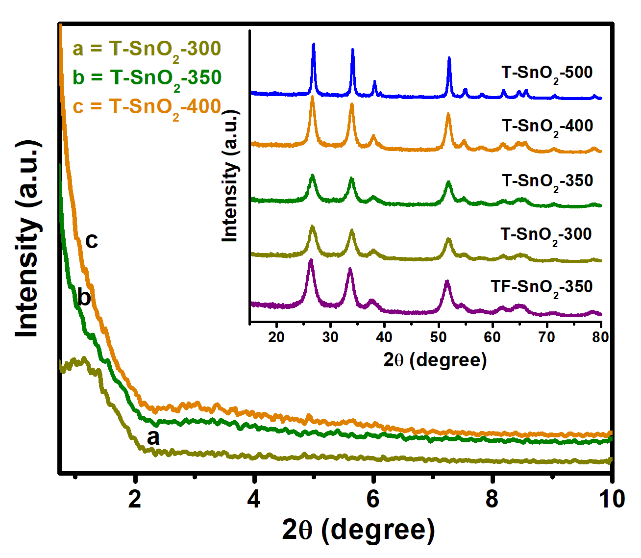


Fig. S1. Low and wide angle XRD patterns of SnO_2_ catalysts calcined at various temperatures.


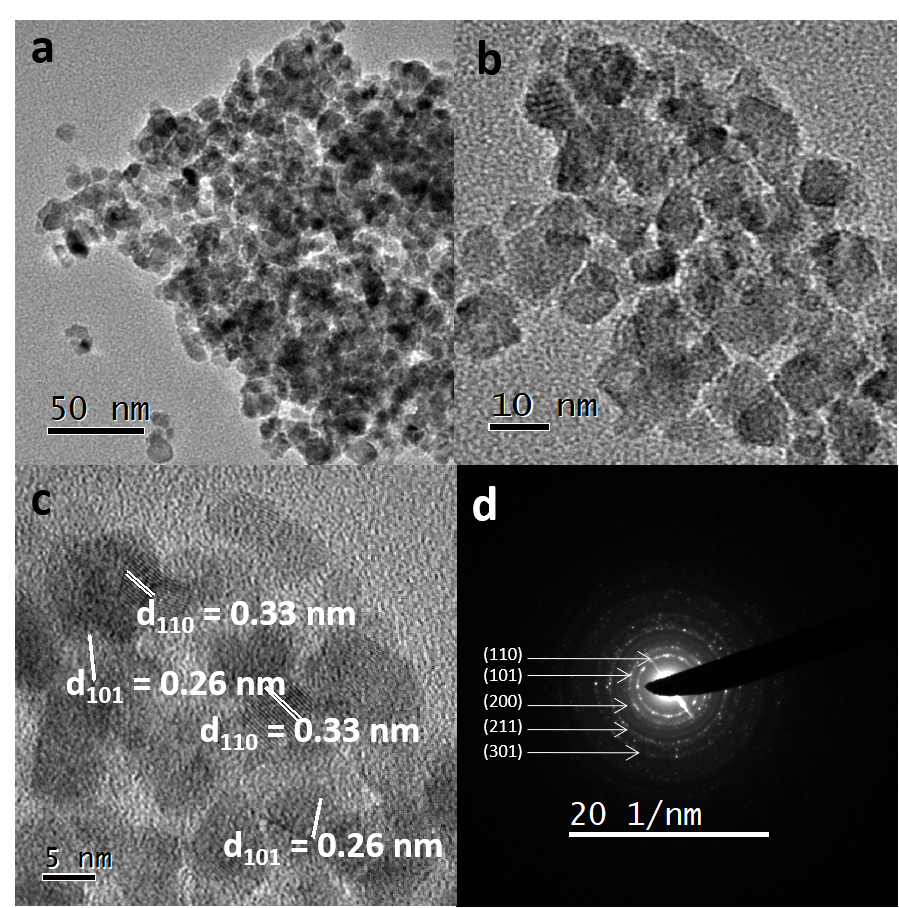


**Fig. S2.** (a and b) TEM image of T-SnO_2_-350; (c) HRTEM image; and (d) SAED images.


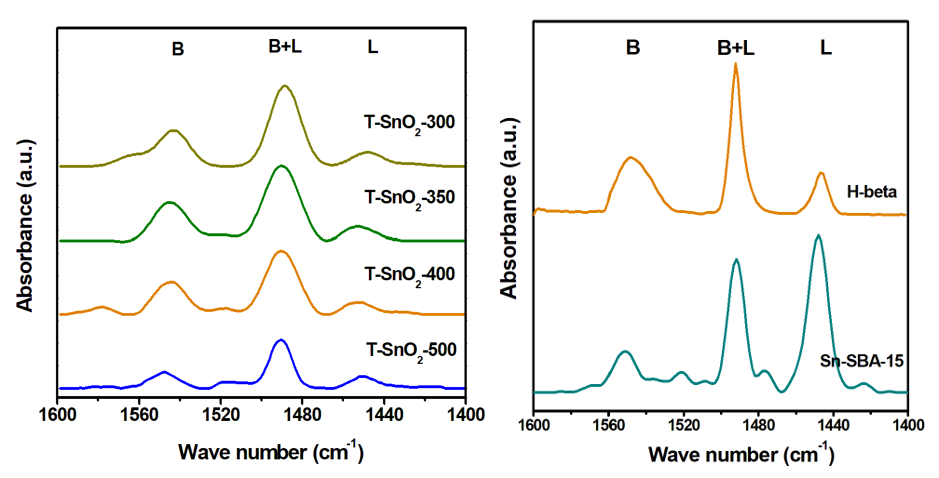


**Fig. S3.** Pyridine FTIR measurement of T-SnO_2_-*x*, H-beta and Sn-SBA-15 catalysts


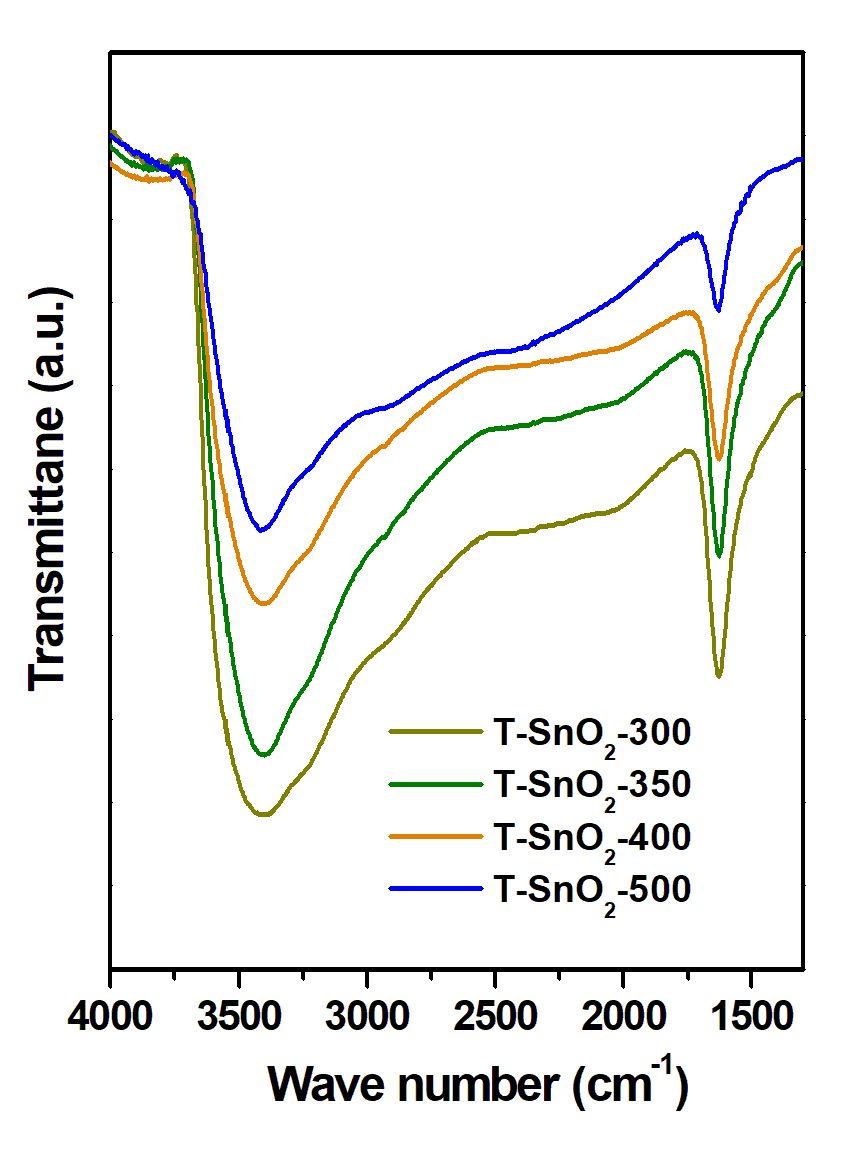


**Fig. S4.** FTIR spectra of T-SnO_2_-*x* catalysts


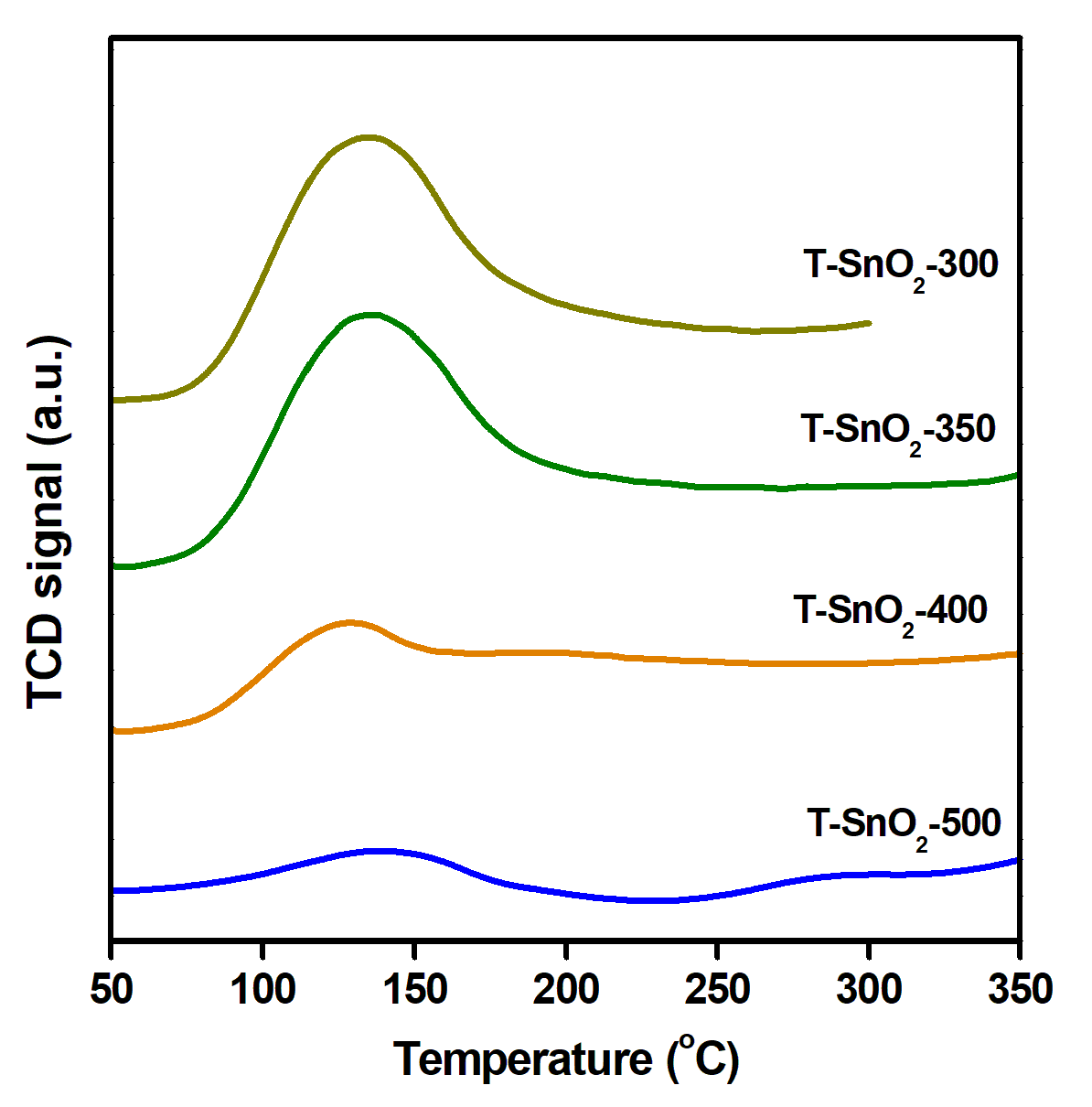


**Fig. S5.** NH_3_-TPD plots of tin oxide catalysts.

**Optimization parameters**

The reaction parameter such as reactant concentration and catalyst amount showed an effect on the yield of 2-methoxy-2-phenylethanol from epoxy styrene and methanol. Firstly, when the reaction was conducted at reflux temperature, the effect of reactant mole concentrations (ratios) from 1:10 to 1:35 was investigated and illustrated in ESI Fig. S6a. The reactant mole ratio had a remarkable effect on the conversion of epoxy styrene with increased concentration. The conversion of epoxy styrene was largely increased from 56 for 1:10 to 100 % for 1:30 with 92 to 96.5% selectivity to 2-methoxy-2-phenylethanol respectively due to the greater effective collisions of the reactants. Furthermore, to optimize, the reaction was performed at optimum reactant mole ratio by varying the catalyst amount from 2.5 to 10 wt% as shown in ESI Fig. S6b. The epoxy styrene conversion and 2-methoxy-2-phenylethanol selectivity increased with increasing the catalyst amount. A maximum of 100% conversion was reached using 7.5 wt% catalyst due to a greater availability of active sites for the reactants and further increase of catalyst to 10 wt% remains with the same activity. While it is expected for the higher catalyst weight of 10% to retain 100% conversion, it was still performed to check if it had any effect on the selectivity. It is seen that the selectivity was largely unaffected by the catalyst concentration beyond 5%. The influence of reaction time at optimum condition (ESI Fig. S7) showed an increase of epoxy styrene conversion from 80% (at 5 minutes) to the maximum 100% (at 30 minutes) with 96.5% selectivity to 2-methoxy-2-phenylethanol.


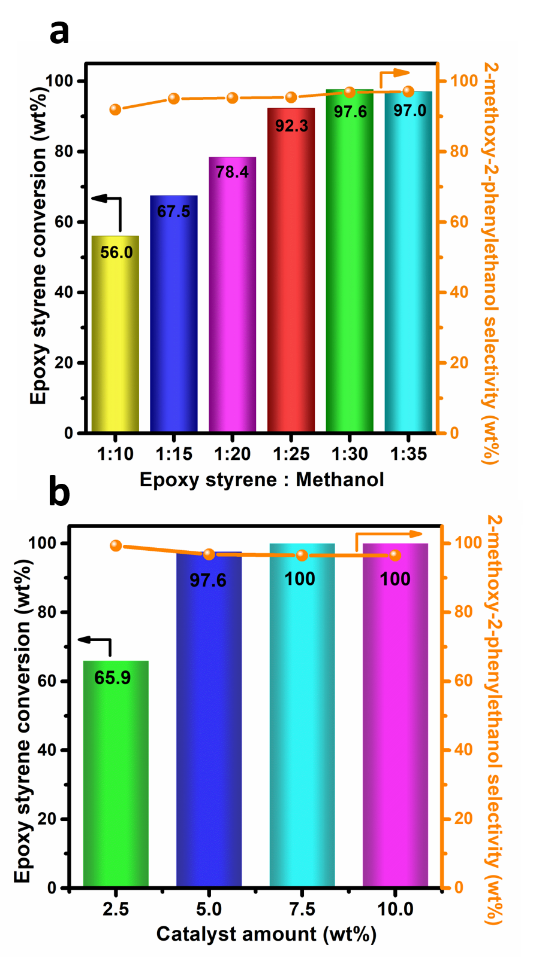


**Fig. S6.** Influence of reaction conditions on epoxy styrene methanolysis over T-SnO_2_-350 catalyst. (a) Effect of reactant mole ratio: Reaction conditions: Epoxy styrene = 2 g, catalyst = 5 wt% wrt epoxy styrene, reaction temp = 65 °C, time = 30 min, (b) Effect of catalyst amount: Reaction conditions: Epoxy styrene = 2 g, methanol = 16 g (Epoxy styrene to methanol mole ratio = 1:30), catalyst = wt% wrt epoxy styrene, reaction temp = 65 °C, time = 30 min.


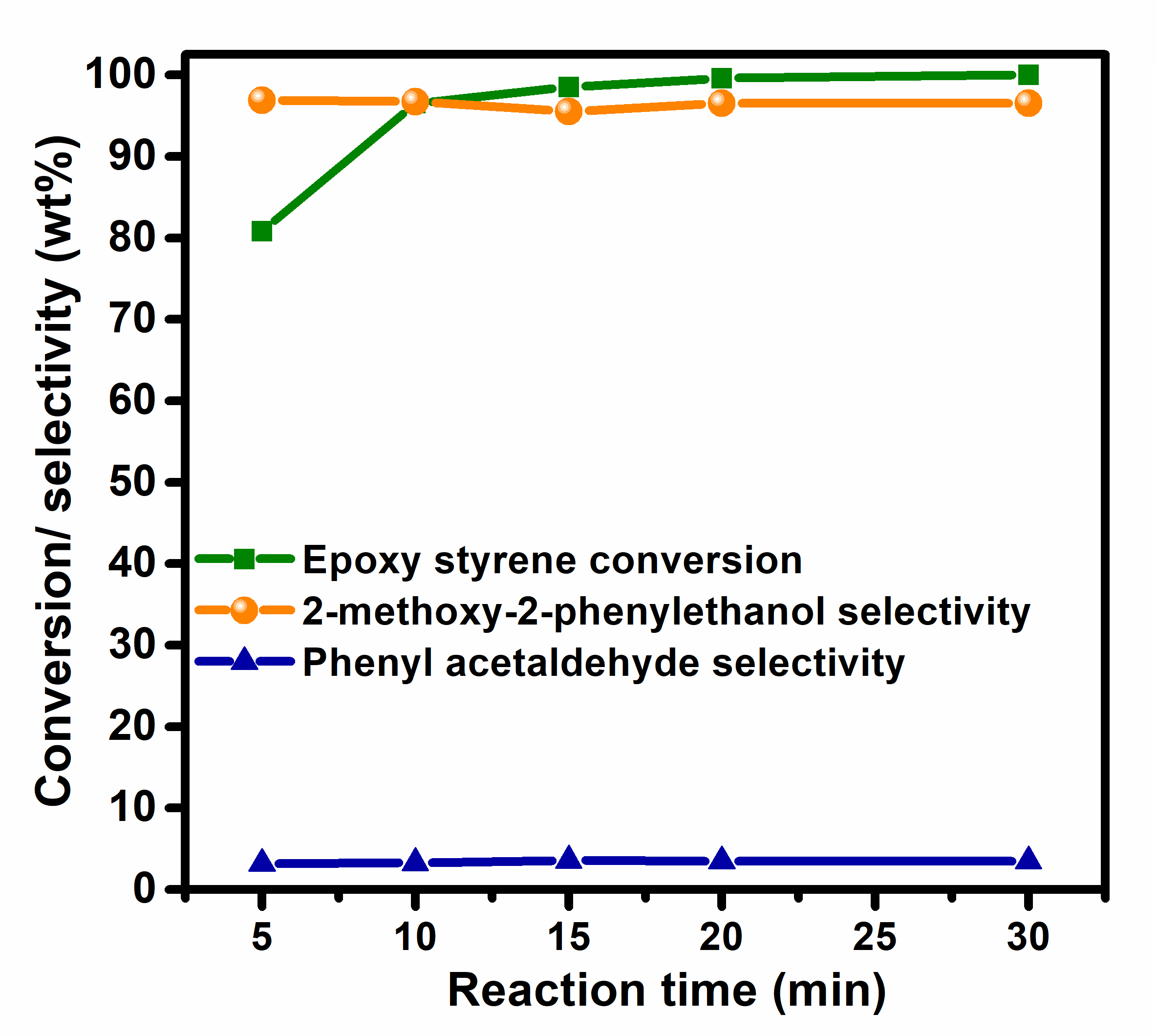


**Fig. S7.** Influence of time on the **c**atalytic activity of T-SnO_2_-350 for alcoholysis of styrene oxide with methanol; Reaction conditions: Epoxy styrene = 2 g, methanol = 16 g, catalyst = 7.5 wt% wrt styrene oxide, reaction temperature = 65 °C, time = 30 min.


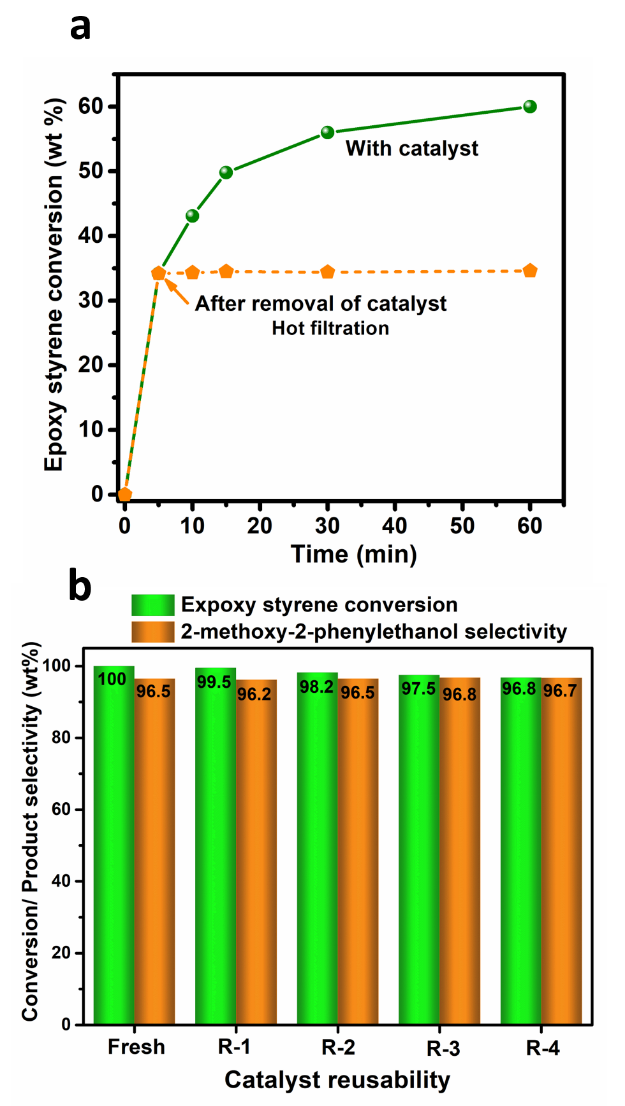


**Fig. S8.** Leaching and catalyst reusability studies of T-SnO_2_-350 catalyst. (a) Conditions: Epoxy styrene = 2 g, methanol = 5.3 g (mole ratio = 1:10), Catalyst = 0.1 g, reaction temp = 65 °C. (b) Conditions: Epoxy styrene : methanol (mole ratio) = 1:30 , Catalyst = 7.5 wt% wrt epoxy styrene, reaction temp = 65 °C, time = 30 min.

**Table S1.** Crystallite size calculations using Scherrer equation

| **Catalyst** | **Crystallite size (nm)** |
| --- | --- |
| TF-SnO_2_-350 | 4.7 |
| T-SnO_2_-300 | 4.4 |
| T-SnO_2_-350 | 4.7 |
| T-SnO_2_-400 | 6.3 |
| T-SnO_2_-500 | 13.8 |

| **Table S2.** Catalytic activity of SnO_2_ catalysts for alcoholysis of epoxy styrene with methanol | | | | | | | |
| --- | --- | --- | --- | --- | --- | --- | --- |
| **Catalysts** | **Total Acidity (μmol NH_3 des_/g)** ^[a]^ | **B/L ratio** ^[b]^ | **Brønsted acidity (μmol H^+^/g)** | **Epoxy styrene conversion (wt%)** | **Product Selectivity (wt%)** | | **A**  **(wt%)** |
|  |  |  |  |  | **A** | **B** |  |
| Blank ^a^ | - |  | - | 0.1 | 100 | - | 0.1 |
| T-SnO_2_-300 | 410 | 4.4 | 334 | 42.1 | 93.2 | 6.8 | 39.2 |
| T-SnO_2_-350 | 440 | 4.0 | 352 | 56.0 | 92.6 | 7.4 | 51.9 |
| T-SnO_2_-400 | 290 | 2.9 | 216 | 30.6 | 92.5 | 7.5 | 28.3 |
| T-SnO_2_-500 | 240 | 1.8 | 154 | 10.3 | 92.2 | 7.8 | 7.7 |
| **^a^ Reaction conditions:** Epoxy styrene = 2 g, methanol = 5.3 g (mole ratio = 1:10), Catalyst = 0.1 g, reaction temp = 65 °C, time = 30 min, [a] time = 60 min, [a] NH_3_-TPD, [b] Py-FTIR, A = 2-Methoxy-2-phenylethanol, B = Phenyl acetaldehyde. | | | | | | | |


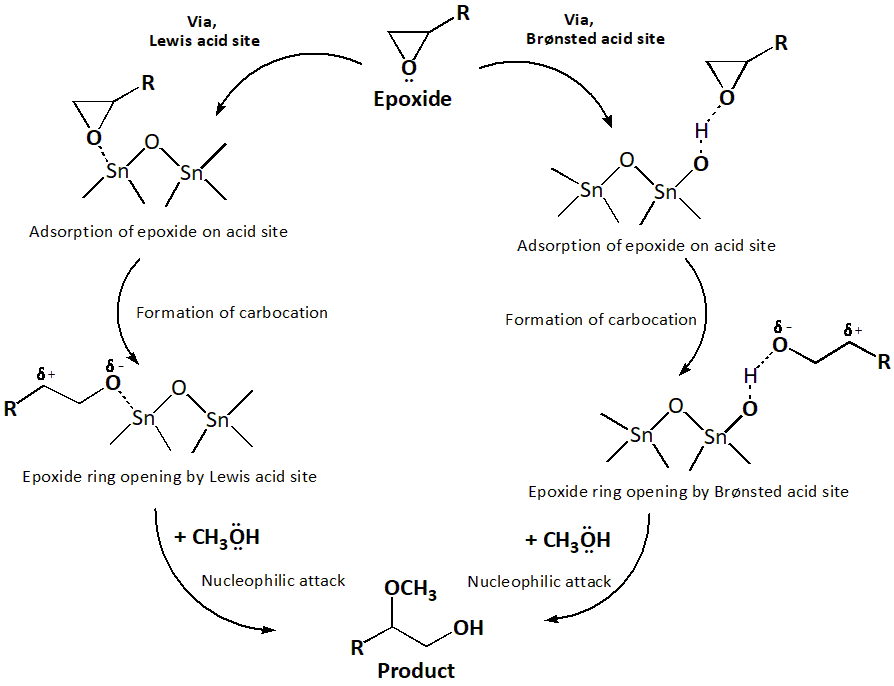


Scheme S1: Plausible mechanism for the ring opening of epoxide in MeOH catalyzed by the Brønsted and Lewis acid sites.

The active site responsible for catalytic activity was identified by 2,6-lutidine studies (Fig. 4) and it revealed that both the Lewis and Brønsted acid sites are responsible for this reaction with the major contribution from Brønsted sites. Based on these results, it is clear that the adsorption and activation of epoxides on Brønsted acid sites is most preferred due to its large availability which provides an easy accessibility to epoxide and enables the ring opening by protonation significantly. This results in an increase of the electrophilic nature of the carbon atom (carbocation) and further it reacts with a nucleophile alcohol to form the product (Scheme S1). Based on these a plausible reaction mechanism is proposed and given in Scheme S1.

**References**

1. Manjunathan P, Maradur SP, Halgeri A, Shanbhag GV. Room temperature synthesis of solketal from acetalization of glycerol with acetone: Effect of crystallite size and the role of acidity of beta zeolite. *Journal of Molecular Catalysis A: Chemical* **396**, 47-54 (2015).

2. Emeis C. Determination of integrated molar extinction coefficients for infrared absorption bands of pyridine adsorbed on solid acid catalysts. *Journal of Catalysis* **141**, 347-354 (1993).

3. Lai F, Yan F, Wang P, Wang S, Li S, Zhang Z. Highly efficient conversion of cellulose into 5-hydroxymethylfurfural using temperature-responsive ChnH5-nCeW12O40 (n= 1–5) catalysts. *Chemical Engineering Journal* **396**, 125282 (2020).

4. Hodala JL, Jung J-S, Yang E-H, Hong GH, Noh YS, Moon DJ. Hydrocracking of FT-wax to fuels over non-noble metal catalysts. *Fuel* **185**, 339-347 (2016).

5. Manjunathan P, Ravishankar R, Shanbhag GV. Novel bifunctional Zn–Sn composite oxide catalyst for the selective synthesis of glycerol carbonate by carbonylation of glycerol with urea. *ChemCatChem* **8**, 631-639 (2016).
